# Efficient Inactivation of Monkeypox Virus by World Health Organization‒Recommended Hand Rub Formulations and Alcohols

**DOI:** 10.3201/eid2901.221429

**Published:** 2023-01

**Authors:** Toni L. Meister, Ronny Tao, Yannick Brüggemann, Daniel Todt, Joerg Steinmann, Joerg Timm, Ingo Drexler, Eike Steinmann

**Affiliations:** Ruhr University Bochum, Bochum, Germany (T.L. Meister, Y. Brüggemann, D. Todt, E. Steinmann);; Heinrich-Heine-University, Düsseldorf, Germany (R. Tao, J. Timm, I. Drexler);; European Virus Bioinformatics Center, Jena, Germany (D. Todt);; Paracelsus Medical University, Nuremberg, Germany (J. Steinmann);; University Hospital of Essen, Essen, Germany (J. Steinmann)

**Keywords:** monkeypox virus, enveloped viruses, viruses, efficient inactivation, World Health Organization, hand rub formulations, alcohols, virucidal activity

## Abstract

Increasing nonzoonotic human monkeypox virus (MPXV) infections urge reevaluation of inactivation strategies. We demonstrate efficient inactivation of MPXV by 2 World Health Organization‒recommended alcohol-based hand rub solutions. When compared with other (re)emerging enveloped viruses, MPXV displayed the greatest stability. Our results support rigorous adherence to use of alcohol-based disinfectants.

The global spread of human monkeypox virus (MPXV) has activated the highest alert level of the World Health Organization (WHO), which declared the virus a public health emergency of international concern (*1*). MPXV is spreading among persons who have not traveled to disease-endemic areas (*2*). The associated novel clinical and epidemiologic patterns necessitate comprehensive investigations, including (re)evaluation of hygiene measures for preventing MPXV transmission. Given the remarkable stability of other pox viruses compared with other enveloped viruses, it is particularly useful to confirm which disinfectants and biocidal agents can inactivate MPXV (*3*). Moreover, in addition to direct contact with infected body fluids or lesions or respiratory secretions, MPXV can be transmitted indirectly through contaminated surfaces (fomites) (*4*). Hygienic hand antisepsis is one of the most useful measures in preventing healthcare- and outbreak-associated viral infections.

In 2009, the WHO proposed Guidelines on Hand Hygiene in Health Care, a document to implement use of 2 alcohol-based hand rubs (formulation I and II) for surgical and hygiene hand disinfection in healthcare settings and to reduce transmission of pathogens (*5*). However, inactivation efficacies of these products against MPVX have not been determined. We evaluated the WHO-recommended alcohol-based formulations against MPXV and performed a comparative inactivation analysis with other (re)emerging enveloped and reference viruses, including Zika virus, influenza A(H1N1) virus, Ebola virus, severe acute respiratory syndrome coronaviruses 1 and 2, and Middle East respiratory syndrome coronavirus.

## The Study

We cultured Vero 76 cells in Dulbecco modified Eagle medium supplemented with 10% (vol/vol) fetal calf serum, 1% (vol/vol) nonessential amino acids, 100 IU/mL penicillin, 100 μg/mL streptomycin, and 2 mmol/L l-glutamine. For preparation of MPXV, we seeded cells at a concentration of 0.33 × 10^6^ cells/mL in a 75-cm^2^ flask in a total volume of 12 mL. The next day, the medium was changed and cells were inoculated with MPXV at a multiplicity of infection of 0.01 and incubated at 37°C until a visible cytopathic effect occurred. Infected cells were harvested by scraping, subjected to 3 freeze/thaw cycles, extensively vortexed and, together with the infectious supernatant purified from cell debris by subsequent centrifugation for 5 min at 1,500 rpm. The virus suspensions were aliquoted, titrated according to standard methods, and stored at −80°C until further use. Virus isolate MPXV-DUS_001 was originally obtained from a patient in Düsseldorf, Germany, who has been infected early during the outbreak 2022, and the isolate was passaged twice on Vero 76 cells before experimental use.

We confirmed the presence of MPXV by a 2-step, quantitative, real-time reverse transcription PCR (qRT-PCR). The first step determines the presence of orthopoxvirus-specific DNA by using panorthopoxvirus‒specific qRT-PCR, and the second step detects and differentiates MPXV clade I (former Congo Basin) and II (former West African) by using an MPXV-specific qRT-PCR. Isolate MPXV-DUS_001 was classified as MPXV clade II.

We assessed virucidal activity of WHO formulation I and II, as well as ethanol and 2-propanol, based on European guideline EN14476 as described (*6*). In brief, we mixed 8 parts of disinfectant with 1 part of interfering substance (bovine serum albumin, final concentration 0.3 g/L) and 1 part MPXV, then vortexed the suspension and incubated for 30 s at room temperature. We then performed an endpoint dilution assay on Vero 76 cells. After 7 days, we evaluated cytopathic effects microscopically and calculated the 50% tissue culture infectious dose per milliliter (Appendix). We tested all 4 disinfectants for final concentrations of 20%, 30%, 40%, 60%. and 80%. 

**Figure 1:**
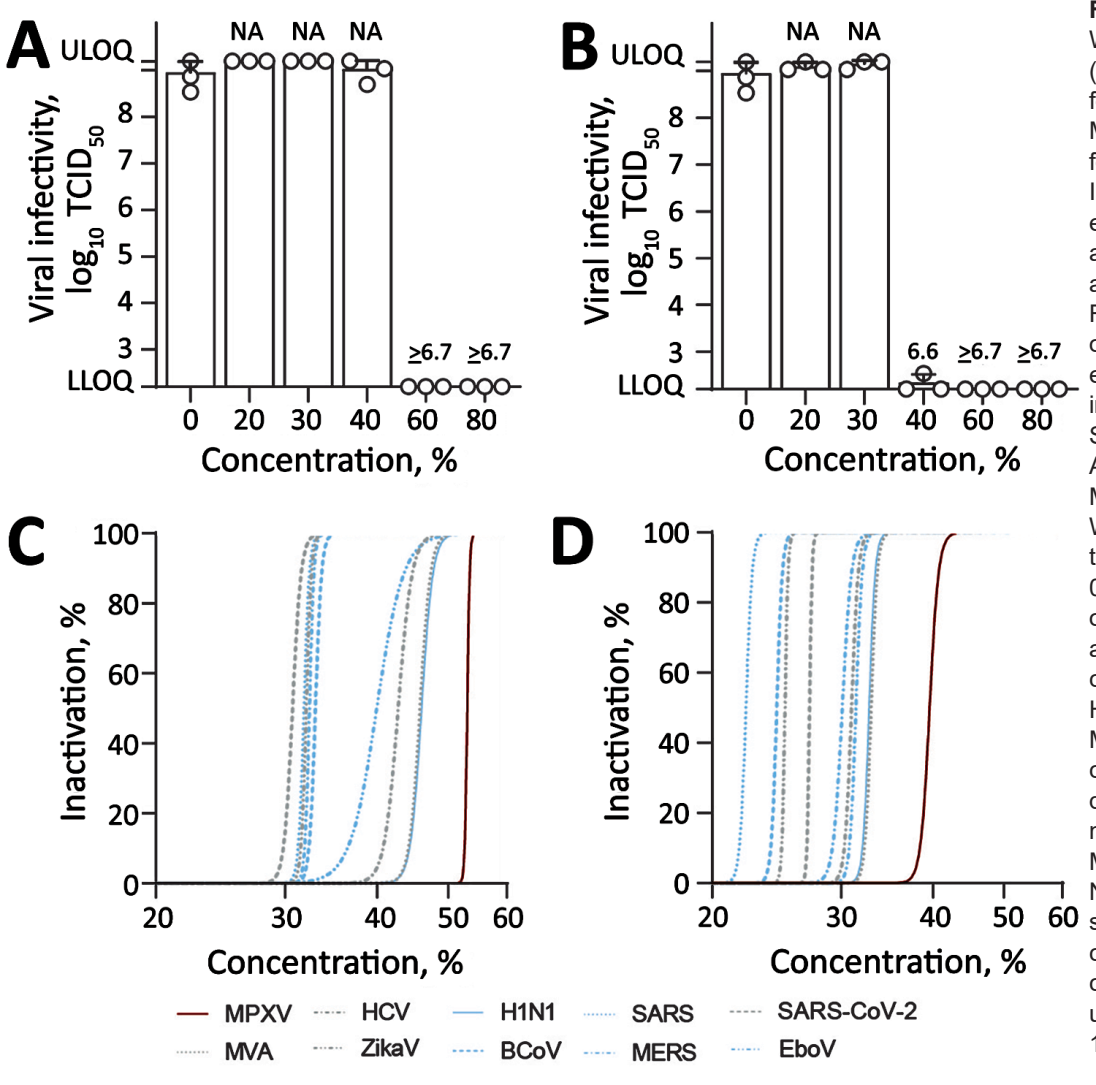
Virucidal activity of World Health Organization (WHO)–recommended hand rub formulations I and II for inactivating MPXV. A, B) Viral infectivity for WHO formulation I (A) and formulation II (B). Means of 3 independent experiments with SDs (error bars) and reduction factors (numbers above the bar) are shown. C, D) Regression analyses of inactivation of MPXV and (re)emerging enveloped or reference viruses, including ZIKV, EBOV, SARS-CoV, SARS-CoV-2, MERS-CoV, influenza A(H1N1) virus, BCoV, HCV, and MVA for WHO formulation I (C) and WHO formulation II (D). Dilutions of the WHO formulations ranging from 0% to 80% with an exposure time of 30 s. Viral titers are displayed as TCID_50_/mL. BCoV, bovine coronavirus; EBOV, Ebola virus; HCV, hepatitis C virus; MERS-CoV, Middle East respiratory syndrome coronavirus; LLOQ, lower limit of quantification (1.58 × 10^2^ TCID_50_/mL); MPXV, monkeypox virus; MVA, modified vaccinia Ankara; NA, not applicable; SARS-CoV2, severe acute respiratory syndrome coronavirus 2; TCID_50_, 50% tissue culture infectious dose; ULOQ, upper limit of quantification (1.58 × 10^9^ TCID_50_/mL); ZIKV, Zika virus.

**Figure 2:**
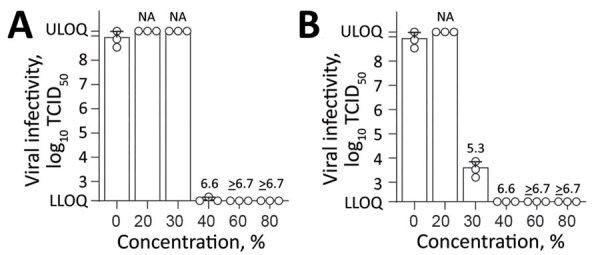
Effect of commercially available alcohols in inactivating monkeypox virus. A) Results for ethanol. B) Results for 2-propanol. Means of 3 independent experiments with SDs (error bars) are shown. Reduction factors are included above the bars. Biocide concentrations ranged from 0% to 80% with an exposure time of 30 s. Viral titers are displayed as TCID_50_/mL values. LLOQ, lower limit of quantification (1.58 × 10^2^ TCID_50_/mL); NA, not applicable; TCID_50_, 50% tissue culture infectious dose; ULOQ, upper limit of quantification (1.58 × 10^9^ TCID_50_/mL).

We examined the virucidal activity of the WHO formulations I and II against MPXV by using a quantitative suspension test. MPXV was highly susceptible to both formulations (Figure 1). The WHO formulation I, based on 80% ethanol (vol/vol), efficiently inactivated the virus with reduction factors (RFs) >6.7 at concentrations of 60% and 80% (vol/vol) (Figure 1, panel A). Likewise, the WHO formulation II, based on 75% isopropanol (vol/vol), inactivated the virus with RFs >6.7 at concentrations of 60% and 80% (vol/vol) (Figure 1, panel B). A dilution of 40% (vol/vol) was still effective for the WHO formulation II with a RF of 6.6, whereas we observed no reduction in viral titer for WHO formulation I at a similar concentration. Subsequent regression analysis of both WHO formulations enabled a quantitative comparison of MPXV inactivation with different other (re)emerging enveloped or reference viruses (Figure 2, panels C, D). Among all viruses, including its reference virus modified vaccinia Ankara, MPXV showed the highest stability against both tested WHO formulations.

Next, we examined the susceptibility of MPXV against the individual components (ethanol and 2-propanol) of both WHO formulations, which are also the main ingredients of numerous commercially available hand disinfections. Both ethanol (Figure 2, panel A) and 2-propanol (Figure 2, panel B) reduced viral titers to background levels with RFs >6.7 at concentrations >60% and >40% (vol/vol), respectively. A minimal concentration of 40% ethanol (vol/vol) nearly inactivated the virus completely (RF 6.6). For 2-propanol, a virucidal activity was observed at a minimal concentration of 30% (vol/vol) with a RF of 5.3.

Overall, both WHO formulations efficiently inactivated MPXV, but the 2-propanol‒based WHO formulation II showed a slightly greater virucidal activity than the ethanol-based WHO formulation I. Nonetheless, MPXV showed the greatest stability to both formulations compared with other (re)emerging enveloped or reference viruses.

## Conclusions

Poxvirus virions are known for their long-term environmental persistence (*3*,*7*). For example, infectious MPXV was recently reported to persist in a household environment for >15 days (*8*). Moreover, because of tight binding with fibrin matrixes of scab/crust material, virions shed from infectious lesion material are even more resistant to desiccation than other enveloped viruses (e.g., influenza virus, rubella virus) (*8**–**10*). Considering the high stability of other poxvirus virions, it is probable that MPXV will share this characteristic, requiring a comprehensive reevaluation of current hygiene measures. 

Poxvirus virions are known for their long-term environmental persistence (*3*,*7*). For example, infectious MPXV was recently reported to persist in a household environment for >15 days (*8*). Moreover, because of tight binding with fibrin matrixes of scab/crust material, virions shed from infectious lesion material are even more resistant to desiccation than other enveloped viruses (e.g., influenza virus, rubella virus) (*8**‒**10*). Considering the high stability of other poxvirus virions, it is probable that MPXV will share this characteristic, requiring a comprehensive reevaluation of current hygiene measures. The WHO recommends 2 inexpensive alcohol-based hand rub formulations to reduce the transmission of pathogens (*5*). We found that MPXV was efficiently inactivated by both formulations, supporting their use in healthcare systems and during MPXV outbreaks.

In addition, ethanol and 2-propanol inactivated the virus in during a 30-s exposure at a concentration of >30% (vol/vol). A comparative inactivation analyses with different (re)emerging enveloped or reference viruses showed that MPXV had the highest stability against both WHO formulations compared with other enveloped viruses. The susceptibility of the different viruses to the WHO formulations probably depends on virus-specific surface properties of their lipophilic envelope. Nonetheless, our results confirm modified vaccinia Ankara as a suitable model surrogate for MPXV to evaluate chemical disinfectants and antiseptics (*11*). WHO formulation II and 2-propanol were slightly more efficient in inactivating MPXV compared with WHO formulation I and ethanol. This difference can probably be explained by the additional carbon of 2-propanol, resulting in an enhanced lipophilicity against the viral membranes compared with ethanol (*12*). Our findings underscore the need and timely application of alcohol-based disinfectants as an effective measure for minimizing viral transmission and maximizing viral inactivation during the ongoing MPVX outbreak. 

Appendix Additional information on efficient inactivation of monkeypox virus by World Health Organization‒recommended hand rub formulations and alcohols.

## References

[1] World Health Organization. WHO Director-General declares the ongoing monkeypox outbreak a public health emergency of international concern [cited 2022 Nov 9]. https://www.who.int/europe/news/item/23-07-2022-who-director-general-declares-the-ongoing-monkeypox-outbreak-a-public-health-event-of-international-concern

[2] World Health Organization. Multi-country monkeypox outbreak in non-endemic countries: update [2022 Nov 9]. https://www.who.int/emergencies/disease-outbreak-news/item/2022-DON388

[3] Wißmann JE, Kirchhoff L, Brüggemann Y, Todt D, Steinmann J, Steinmann E. Persistence of pathogens on inanimate surfaces: a narrative review. Microorganisms. 2021;9:343. 10.3390/microorganisms9020343 33572303 PMC7916105

[4] Brown K, Leggat PA. Human monkeypox: current state of knowledge and implications for the future. Trop Med Infect Dis. 2016;1:E8. 10.3390/tropicalmed1010008 30270859 PMC6082047

[5] World Health Organization. WHO guidelines on hand hygiene in health care: first global patient safety challenge clean care is safer care. Geneva: The Organization; 2009. 23805438

[6] Kratzel A, Todt D, V’kovski P, Steiner S, Gultom M, Thao TTN, et al. Inactivation of severe acute respiratory syndrome coronavirus 2 by WHO-recommended hand rub formulations and alcohols. Emerg Infect Dis. 2020;26:1592–5. 10.3201/eid2607.200915 32284092 PMC7323537

[7] Kampf G. Efficacy of heat against the vaccinia virus, variola virus and monkeypox virus. J Hosp Infect. 2022;127:131–2. 10.1016/j.jhin.2022.06.008 35728695 PMC9582962

[8] Morgan CN, Whitehill F, Doty JB, Schulte J, Matheny A, Stringer J, et al. Environmental persistence of monkeypox virus on surfaces in household of person with travel-associated infection, Dallas, Texas, USA, 2021. Emerg Infect Dis. 2022;28:1982–9. 10.3201/eid2810.221047 35951009 PMC9514334

[9] Nolen LD, Osadebe L, Katomba J, Likofata J, Mukadi D, Monroe B, et al. Introduction of monkeypox into a community and household: risk factors and zoonotic reservoirs in the Democratic Republic of the Congo. Am J Trop Med Hyg. 2015;93:410–5. 10.4269/ajtmh.15-0168 26013374 PMC4530773

[10] Hutson CL, Carroll DS, Gallardo-Romero N, Weiss S, Clemmons C, Hughes CM, et al. Monkeypox disease transmission in an experimental setting: prairie dog animal model. PLoS One. 2011;6:e28295. 10.1371/journal.pone.0028295 22164263 PMC3229555

[11] Rabenau HF, Rapp I, Steinmann J. Can vaccinia virus be replaced by MVA virus for testing virucidal activity of chemical disinfectants? BMC Infect Dis. 2010;10:185. 10.1186/1471-2334-10-185 20573218 PMC2908096

[12] Schürmann W, Eggers HJ. Antiviral activity of an alcoholic hand disinfectant. Comparison of the in vitro suspension test with in vivo experiments on hands, and on individual fingertips. Antiviral Res. 1983;3:25–41. 10.1016/0166-3542(83)90012-8 6870229

