# Supplementary material for: Efficient Inactivation of Monkeypox Virus by World Health Organization‒Recommended Hand Rub Formulations and Alcohols
Source: Emerg Infect Dis. 2023 Jan;29(1):189–92. doi: 10.3201/eid2901.221429 (PMC9796202; doi:10.3201/eid2901.221429)
Supplement: Appendix — Additional information on efficient inactivation of monkeypox virus by World Health Organization‒recommended hand rub formulations and alcohols. [file 22-1429-Techapp-s1.pdf]

Article DOI: https://doi.org/10.3201/eid2901.221429

Efficient Inactivation of Monkeypox Virus by World Health Organization–Recommended Hand Rub Formulations and Alcohols

Appendix

Statistical Analyses

Dose-response curves were determined as percent normalized virus inactivation versus percent log disinfectant concentration by nonlinear regression by using a robust fitting method on normalized 50% tissue culture infectious dose data implemented in Prism version 9.4.1 (GraphPad, https://www.graphpad.com) and reduction factors were determined as described (*1*). Reference curves for comparative (re)emerging enveloped and reference viruses were generated in previously published studies (*1*,*2*). Because of broad concentration steps of World Health Organization formulations, these curves do not necessarily represent the exact inactivation course, but rather give an impression of monkeypox virus sensitivity in the context of reference viruses.

References

1. Kratzel A, Todt D, V'kovski P, Steiner S, Gultom M, Thao TT, et al. Inactivation of severe acute respiratory syndrome coronavirus 2 by WHO-recommended hand rub formulations and alcohols. Emerg Infect Dis. 2020;26:1592–5. PubMed https://doi.org/10.3201/eid2607.200915

2. Siddharta A, Pfaender S, Vielle NJ, Dijkman R, Friesland M, Becker B, et al. Virucidal activity of World Health Organization-recommended formulations against enveloped viruses, including Zika, Ebola, and emerging coronaviruses. J Infect Dis. 2017;215:902–6. PubMed https://doi.org/10.1093/infdis/jix046
